# Systemic oxidative stress and cognitive function in Parkinson’s disease with different PWMH or DWMH lesions

**DOI:** 10.1186/s12883-020-02037-z

**Published:** 2021-01-11

**Authors:** Ta-Chih Chang, Yi-Cun Chen, Yu-Chi Huang, Wei-Che Lin, Cheng-Hsien Lu

**Affiliations:** 1 Department of Physical Medicine and Rehabilitation, Kaohsiung Chang Gung Memorial Hospital and Chang Gung University College of Medicine, Kaohsiung, Taiwan; 2 Department of Diagnostic Radiology, Kaohsiung Chang Gung Memorial Hospital and Chang Gung University College of Medicine, Kaohsiung, Taiwan; 3 Department of Neurology, Kaohsiung Chang Gung Memorial Hospital and Chang Gung University College of Medicine, Kaohsiung, Taiwan

**Keywords:** Oxidative stress, Cognitive function, Parkinson’s disease, White matter injury

## Abstract

**Background:**

Parkinson’s disease (PD), frequently accompanied by cognitive impairments, is associated with systemic oxidative stress and abnormal structural changes on brain images. We aimed to identify the correlation between systemic oxidative stress and cognitive function in PD patients with different periventricular white matter hyperintensities (PWMH) and deep white matter hyperintensities (DWMH).

**Methods:**

A total of 146 participants with idiopathic PD underwent brain MRI, which revealed PWMH and DWMH. The number of lesions were evaluated using the Fazekas criteria. Systemic oxidative stress was determined as early or late phase changes in leukocyte apoptosis and its subsets by flow cytometry. Cognitive functions, including attention, executive function, memory, language, and visual space, were assessed.

**Results:**

For different DWMH, the leukocyte apoptosis and its subsets were significantly different.. However, there were no significant differences in oxidative stress biomarkers in PD patients with different PWMH. Attention and memory were significantly decreased in patients with more advanced DWMH injuries. Attention, memory, and language were significantly impaired in patients with worse PWMH lesions.

**Conclusion:**

Significant oxidative stress biomarker alternations in PD patients with DWMH, but not PWMH, might be associated with white matter injury. Systemic inflammatory responses may contribute to deep white matter damage in PD. Further, more cognitive deficits were seen in PD patients with worse deep white matter lesions, especially in moderate to severe periventricular white matter injury.

**Trial registration:**

Retrospective study.

## Background

Parkinson’s disease (PD), which is frequently accompanied by cognitive dysfunction, is one of the most common neurodegenerative disorders in the elderly add reference 1 [1]. In PD, loss of neurons occurs in the substantia nigra and in the nigrostriatal pathway to the striatum, along with dopamine depletion [2]. Dopamine depletion from the basal ganglia, leading to disruption of the connections between the thalamus and motor cortex, is related to motor symptoms in PD [3]. In addition to motor dysfunction in PD, dopaminergic deficiency in interrupting the modulation of fronto-striatal networks is associated with cognitive impairment [4]. Although the clear pathogenic mechanisms of neurodegeneration in PD are not yet understood, they may involve a cascade of events that include interactions between genetic and environmental factors [5], abnormalities in protein processing [6], immune regulation [7], mitochondrial dysfunction [8], inflammation [9], and oxidative stress [10].

Increased systemic oxidative is associated with neuroinflammation in PD and progression of disease through various pathways, including blood-brain barrier dysfunction, microglia activation, and infiltration of peripheral immune cells and circulating cytokines [7, 9, 11]. Increased oxidative stress has also been reported to be associated with cognitive impairment in PD. [12, 13] The infiltration of peripheral leucocyte apoptosis and peripheral leucocyte adhesion molecules was found to be one of the interactive pathways between systemic oxidative stress and neuroinflammation [14]. Furthermore, in patients with PD, leucocyte apoptosis was significantly high and was associated with striatal dopamine neuron loss, as determined by magnetic resonance imaging (MRI) [15]. This evidence suggests that peripheral immune cells, which reflect the level of systemic oxidative stress, might play important roles in the development of PD and cognitive impairment.

Brain white matter is vulnerable to oxidative stress due to its relatively low intrinsic antioxidative properties [16]. White matter hyperintensities (WMHs), also called leucoaraiosis or white matter lesions [17], are frequently observed on brain MRI scans in elderly individuals [18], and higher WMH burdens are associated with worse cognitive performance in the general population, patients with Alzheimer’s disease, and patients with PD. [19–21] In addition, systemic inflammation was found to be associated with white matter damage in patients with PD [14], obstructive sleep apnoea [22], and several neurological diseases [23]. WMH was reported to be an important risk factor for PD with mild cognitive impairment (PD-MCI) [24], although the evidence was still conflicting and inconsistent [25]. WMH can be defined as periventricular white matter hyperintensities (PWMH) and deep white matter hyperintensities (DWMH) [26] by visual rating scales used in clinical settings [27]. This distinction seems to reflect different functional, histopathological, and aetiological features [28, 29]. Previous studies found that the elderly with PWMH, rather than DWMH, are associated with impaired cognitive function, especially in the executive function/processing speed domain, than the memory domain [26, 30]. For PD-MCI patients, one study demonstrated that PWMH was associated with worse cognitive performance, specifically, executive impairment and visuospatial impairment [21].

The objectives of our study were (1) to investigate the associations of systemic oxidative stress biomarkers in PD patients with DWMH or PWMH and (2) to identify cognitive performance in PD patients with different classifications or levels of white matter injuries.

## Methods

### Participants

In this retrospective study, we recruited one hundred and forty-six idiopathic PD patients who participated in the previous trials conducted from January, 2011 to June, 2018. All these previous trials investigated the neuropsychological functions, oxidative stress, and brain MRI findings in the patients with PD. Idiopathic PD was diagnosed by experienced neurologists according to the United Kingdom Brain Bank criteria [31]. Patients with a history of other neurological or psychiatric disorders were excluded from the study. For each patient, the physical and neurological examinations and imaging assessments were all recorded in the ON-medication state. The Chang Gung Medical Foundation Institutional Review Board approved the study and previous trials, and all of the participants or their guardians provided written informed consent, including this study and previous trials. This study was funded by NMRPG8J6022 (MOST 108–2314-B-182A-014-MY3), NMRPG8J0271 (MOST 108–2314-B-182A-017), and CMRPG8K0221.

### Disease severity and neuropsychological assessments

For each patient with idiopathic PD, disease severity and functional condition were reported according to the results of the Unified Parkinson’s Disease Rating Scale (UPDRS) [32], the modified Hoehn and Yahr Staging Scale (HYSS) [33], and the Schwab & England Activities of Daily Living Scale (SE-ADLS) [34]. The sections of UPDRS consist of the following evaluations: mentation, behaviour, and mood and daily activities of speech, salivation, swallowing, handwriting, cutting food and handling utensils, dressing, hygiene, turning in bed, falling, freezing while walking, walking, tremor, sensory complaints, and motor capability. Higher scores represent more severe conditions in patients with PD. The modified HYSS was used to evaluate the severity of PD based on clinical presentations and functional ability from stages 1 to 5 (higher levels indicate higher severity of the disease). SE-ADLS was used to assess a person’s daily function for PD, in which a score of 100% indicates complete independence and 0% indicates a bedridden status with vegetative functions. The Mini-Mental State Examination (MMSE) was used to assess cognitive function in patients with idiopathic PD. Additionally, the subtests from the Cognitive Ability Screening Instrument (CASI) [35] and the Wechsler Adult Intelligence Scale-III (WAIS-III) [36] were used to build a neuropsychological battery including five domains (attention, executive function, language, memory, and visuospatial function) [37]. A neuropsychological battery was performed by a clinical psychologist blinded to each patient’s status. The attention function consisted of the digit span score from the WAIS-III and the attention and orientation scores from the CASI. Executive functions included the similarity, arithmetic, matrix reasoning, picture arrangement, and digit symbol coding scores in the WAIS-III and the abstract thinking and judgement score in the CASI. Speech and language functions were presented using the vocabulary and comprehension scores in the WAIS-III and the language score in the CASI. Memory function was assessed using the information score of the WAIS-III and the short- and long-term memory scores of the CASI, while the visuo-spatial function was assessed using the picture completion and block design scores from the WAIS-III and the drawing score from the CASI. Furthermore, these five cognitive domains were analysed with the average Z-score of all subtest scores.

### MRI acquisition

MRI scans were acquired on a GE Signa 3 T whole-body MRI scanner (General Electric Healthcare, Milwaukee, WI, USA) using an eight-channel phase array head coil at the Kaohsiung Chang Gung Memorial Hospital in Taiwan. Whole brain 3-D T1 weighted images were collected for each participant using an inversion-recovery fluid-attenuated fast spoilt gradient-recalled echo pulse sequence with the following imaging parameters: repetition time (TR)/ echo time (TE)/inversion time (TI) = 9.5/3.9/450 ms; flip angle = 20°; number of excitations (NEX) = 1; field of view (FOV) = 240 × 240 mm^2^; matrix size = 512 × 512; voxel size = 0.47 × 0.47 × 1.3 mm^3^; and slice number = 110 axial slices (without interslice gaps). T2-weighted fast spin-echo sequence (TR/TE = 4200/102 ms; echo train length = 18; NEX = 2; FOV = 240 mm^2^; slice thickness = 5 mm; matrix size = 320 × 256 and 18 slices) and axial fluid-attenuated inversion-recovery sequence (FLAIR) (TR/TE/TI = 8000/100/2000 ms; NEX = 1; FOV = 240 mm^2^; slice thickness = 5 mm; matrix size = 320 × 256 and 18 slices) in the same imaging session.

We applied the Fazekas criteria [38–40] to define the gradings of the PWMH and DWMH scales on brain MRI scans. It described the severity of white matter lesions according to the size and confluence of lesions on the MRI scans; 0 indicated an absent lesion on the images. PWMH scale grading was from 0 to 3: 0 indicated an absent lesion; 1 indicated a “caps” or pencil-thin lining lesion; 2 indicated a smooth “halo” lesion; and 3 indicated an irregular periventricular signal lesion extending into the deep white matter. DWMH scale grading was from 0 to 3: 0 indicated an absent lesion; 1 indicated a punctate foci lesion; 2 indicated the beginning of a confluence lesion; and 3 indicated a large confluent area lesion.

PD patients with PWMH or DWMH were divided into three groups according to the severity of white matter lesions (grade 0: normal, grade 1: mild, and grade 2–3: moderate to severe white matter injuries).

### Blood sampling and assessment of systemic oxidative stress biomarkers

All participants underwent blood sampling by venepuncture while the neuropsychological assessments and brain MRI evaluations were performed simultaneously. In this study, the percentage of peripheral leucocyte apoptosis was used to assess the oxidative stress. A detailed description of the assessment of leucocyte apoptosis has been presented in previous studies [14, 22].

The status of leucocyte apoptosis was assessed with APO 2.7-phycoerythrin (PE) (clone 2.7A6A3; Immunotech) to identify early and late apoptosis. Positive expression of APO 2.7-PE appears to be restricted to cells undergoing apoptosis. The presence of early apoptotic cells indicated that the apoptotic process was reversible in the early stage, but the presence of late apoptotic cells demonstrated that the cell membrane integrity was disrupted. Leucocytes and their subtypes were analysed according to the intensity of CD45 expression using flow cytometry. Results are expressed as a percentage of specific fluorescence-positive cells. Apoptotic cells were defined as those positive for APO 2.7. A database coordinator monitored all data collection and entry, both of which were checked for any inconsistencies.

### Statistical analysis

Age, UPDRS, modified HYSS, SE-ADLS, PWMH grading, DWMH grading, and MMSE were analysed using frequency distribution. The Kruskal-Wallis H test was used for group comparisons of the blood test data in these three groups, and the Mann-Whitney U test was used for between-group comparisons. The scores of each neuropsychological assessment were first changed into Z scores and were further calculated based on five main cognitive categories: attention function, executive function, memory, speech and language, and visuospatial function, and the total of each score represented the ability to perform these psychological functions. Statistical significance was defined as a *p* value < 0.05. All statistical tests were performed using SPSS 19.0 (SPSS, Inc., Chicago, IL, USA).

## Results

### Clinical characteristics in PD patients with white matter lesions

The clinical characteristics of patients with idiopathic PD are presented in Table 1. A total of 146 patients with PD, including 64 males and 82 females, were enrolled in this study. The mean age of the participants was 63.68 years. The percentages of hypertension, diabetes mellitus, hypercholesterolemia and smoking were 40, 13, 21.2 and 2.7%, respectively. The percentage of medications use that possible interfere with cognitive performance, which defined as Benzodiazepines (BZD), was 35.6%. The mean total scores of UPDRS were 38.54, the mean stages in modified HYSS, the SE-ADLS, and MMSE were 1.94, 82.96, and 17.91, respectively. The mean grading of the PWMH and DWMH were 1.16 and 1.12, respectively.

**Table 1:** Clinical characteristics in PD

	PD (***n***=146)
Age (years, mean (SD))	63.68 (10.69)
Sex (male, female)	64;82
HTN (number, percentage)	54 (40%)
DM (number, percentage)	19 (13%)
Hypercholesterolemia (number, percentage)	31 (21.2%)
Smoking (number, percentage)	4 (2.7%)
Medications (number, percentage)	52 (35.6%)
UPDRS I, mean (SD)	3.41 (2.47)
UPDRS II, mean (SD)	10.21 (7.81)
UPDRS III, mean (SD)	24.91 (16.37)
UPDRS total, mean (SD)	38.54 (24.92)
Modified Hoehn and Yahr scale, mean (SD)	1.94 (1.14)
Schwab and England ADL scale, mean (SD)	82.96 (17.91)
PWMH grading, mean (SD)	1.16 (0.59)
DWMH grading, mean (SD)	1.12 (0.66)
MMSE, mean (SD)	24.42 (4.92)

Among the clinical characteristics, age, UPDRS, Hoehn and Yahr scale, Schwab and England ADL scale, PWMH grading, DWMH grading and MMSE were analysed using frequency distribution

*UPDRS* The Unified Parkinson’s Disease Rating Scale, *ADL* Activity of daily living, *PWMH* Periventricular white matter hyperintensity, *DWMH* Deep white matter hyperintensity, *MMSE* Mini-Mental State Examination, *HTN* Hypertension, *DM* Diabetes Mellitus; Medications, was defined as Benzodiazepines

### Group comparisons of disease severity in PD patients with different DWMH or PWMH gradings

According to DWMH or PWMH gradings, 146 participants were divided into three groups (normal, mild, and moderate to severe) depending on white matter alternations on brain images. In the DWMH groups (Table 2), 24, 80, and 42 patients with PD were grouped based on the presence of normal, mild, and moderate to severe white matter lesions (grade 0, 1, 2–3). In the PWMH groups (Table 3), there were 16, 90, and 40 patients with normal (grade 0), mild (grade 1), or moderate to severe (grade 2–3) white matter lesions. There was a significant difference in age among PD patients with different PWMH and DWMH gradings. In DWMH, there was a significant difference in hypertension between mild and moderate to severe white matter lesions. In PWMH, there was significant difference in medications use among different gradings. No significant differences were revealed in gender, total UPDRS, Modified HYSS, and SE-ADLS among the groups with different PWMH and DWMH gradings.

**Table 2:** Disease severity and neuropsychological assessment in PD patients with different DWMH gradings

DWMH gradings	Normal (*n*=24)	Mild (*n*=80)	Moderate to Severe (*n*=42)	P	P_1_	P_2_	P_3_
Gender (male, female)	11,13	30,50	23,19	0.185	0.484	0.610	0.084
Age (years, mean (SD))	55.37 (12.99)	63.06 (9.85)	69.61 (6.72)	0.001*	0.013*	0.001*	0.001*
HTN (number, percentage)	8 (33.3%)	25 (31.3%)	21 (50%)	0.117	0.848	0.193	0.043*
DM (number, percentage)	3 (12.5%)	10 (12.5%)	6 (14.3%)	0.959	1.000	0.840	0.782
Hypercholesterolemia (number, percentage)	3 (12.5%)	16 (20%)	12 (28.6%)	0.286	0.407	0.137	0.287
Smoking (number, percentage)	1 (4.2%)	3 (3.8%)	0	0.436	0.926	0.186	0.206
Medications (number, percentage)	8 (33.3%)	30 (37.5%)	14 (33.3%)	0.873	0.711	1.000	0.650
UPDRS total, mean (SD)	33.91 (23.26)	39.78 (23.05)	38.71 (29.19)	0.458	0.226	0.646	0.478
Modified Hoehn and Yahr scale, mean (SD)	1.71 (0.85)	1.92 (1.09)	2.09 (1.31)	0.706	0.674	0.404	0.583
Schwab and England ADL scale mean (SD)	87.36 (12.40)	82.89 (16.55)	81.00 (22.16)	0.583	0.281	0.528	0.741
MMSE, mean (SD)	25.47 (3.31)	25.32 (4.53)	22.14 (5.71)	0.002*	0.471	0.021*	0.001*
Neuropsychological battery							
Attention, mean (SD)	−0.24 (1.95)	−0.20 (2.03)	−1.57 (2.91)	0.020*	0.899	0.060	0.006*
Executive function, mean (SD)	−1.31 (4.48)	− 0.60 (4.43)	−2.04 (4.69)	0.338	0.441	0.758	0.149
Memory, mean (SD)	0.09 (1.96)	−0.34 (2.17)	−1.36 (2.77)	0.040*	0.620	0.028*	0.027*
Language, mean (SD)	−0.54 (1.73)	−0.26 (2.43)	−1.30 (2.87)	0.127	0.431	0.298	0.051
Visual-spatial, mean (SD)	−1.29 (1.79)	−0.56 (2.12)	−1.26 (2.67)	0.148	0.087	0.714	0.159

Kruskal-Wallis H test was used for between-group comparisons of the three groups, and Mann-Whitney U test was used for between-two-group comparisons

*P* Comparison of group 0, 1, and 2+ 3, *P1* Comparison of group 0 and 1, *P2* Comparison of group 0 and 2+ 3, *P3* Comparison of group 1 and 2+ 3, *SD* Standard deviation, *PD* Parkinson’s disease, *DWMH* Deep white matter hyperintensity, *HTN* Hypertension, *DM* Diabetes Mellitus; Medications, was defined as Benzodiazepines

*p< 0.05

**Table 3:** Disease severity and neuropsychological assessment in PD patients with different PWMH gradings

PWMH gradings	Normal (*n*=16)	Mild (*n*=90)	moderate to severe (*n*=40)	P	P_1_	P_2_	P_3_
Gender (male, female)	9,7	37,53	19, 21	0.523	0.285	0.442	0.704
Age (years, mean (SD))	57.81 (11.49)	62.58 (10.67)	69.50 (8.60)	0.001*	0.067	0.001*	0.001*
HTN (number, percentage)	4 (25%)	31 (34.4%)	19 (47.5%)	0.211	0.461	0.125	0.160
DM (number, percentage)	2 (12.5%)	11 (12.2%)	6 (15%)	0.909	0.975	0.811	0.666
Hypercholesterolemia (number, percentage)	2 (12.5%)	20 (22.2%)	9 (22.5%)	0.665	0.379	0.399	0.972
Smoking (number, percentage)	0	4 (4.4%)	0	0.281	0.392	1.000	0.177
Medications (number, percentage)	11 (68.8%)	28 (31.1%)	13 (32.5%)	0.014*	0.004*	0.014*	0.875
UPDRS total, mean (SD)	43.43 (22.11)	36.68 (23.50)	41.58 (29.09)	0.456	0.229	0.486	0.555
Modified Hoehn and Yahr scale, mean (SD)	2.18 (1.34)	1.86 (1.04)	2.03 (1.26)	0.761	0.445	0.669	0.803
Schwab and England ADL scale, mean (SD)	82.67 (13.87)	83.97 (16.30)	80.52 (22.29)	0.823	0.527	0.680	0.841
MMSE, mean (SD)	27.62 (2.24)	24.71 (4.54)	22.56 (5.73)	0.001*	0.007*	0.001*	0.033*
Neuropsychological battery							
Attention, mean (SD)	0.83 (1.16)	−0.46 (2.17)	−1.44 (2.80)	0.003*	0.030*	0.001*	0.034*
Executive function, mean (SD)	0.71 (3.97)	−0.97 (4.23)	−2.23 (5.11)	0.113	0.130	0.060	0.225
Memory, mean (SD)	0.78 (1.14)	−0.36 (2.06)	−1.54 (2.94)	0.003*	0.033*	0.001*	0.037*
Language, mean (SD)	0.31 (2.12)	−0.36 (2.20)	−1.48 (3.00)	0.040*	0.380	0.044*	0.028*
Visual-spatial, mean (SD)	0.18 (1.96)	−0.83 (2.10)	−1.35 (2.57)	0.058	0.065	0.029*	0.210

Kruskal-Wallis H test was used for between-group comparisons of three groups, and Mann-Whitney U test was used for between-two-group comparisons

*P* Comparison of group 0, 1 and 2+ 3, *P1* Comparison of group 0 and 1, *P2* Comparison of group 0 and 2+ 3, *P3* Comparison of group 1 and 2+ 3, *SD* Standard deviation, *PD* Parkinson’s disease, *PWMH* Periventricular white matter hyperintensities, *HTN* Hypertension, *DM* Diabetes Mellitus; Medications, was defined as Benzodiazepines

*p< 0.05

### Group comparisons of cognitive functions in PD patients with different PWMH or DWMH gradings

There were significant differences in MMSE scores (*p*=0.002 and *p*=0.001) among the three groups with different DWMH gradings and those with different PWMH gradings. There were significant differences in attention function (*p*=0.02) and memory (*p*=0.04) among PD patients with different DWMH gradings (Table 2). In PD patients with different PWMH gradings (Table 3), there were statistical significances in the attention, memory, and language domains of cognition functions (*p*=0.003, 0.003, and 0.04).

### Group comparisons of oxidative stress biomarkers in PD patients with different PWMH or DWMH gradings

**Fig. 1:**
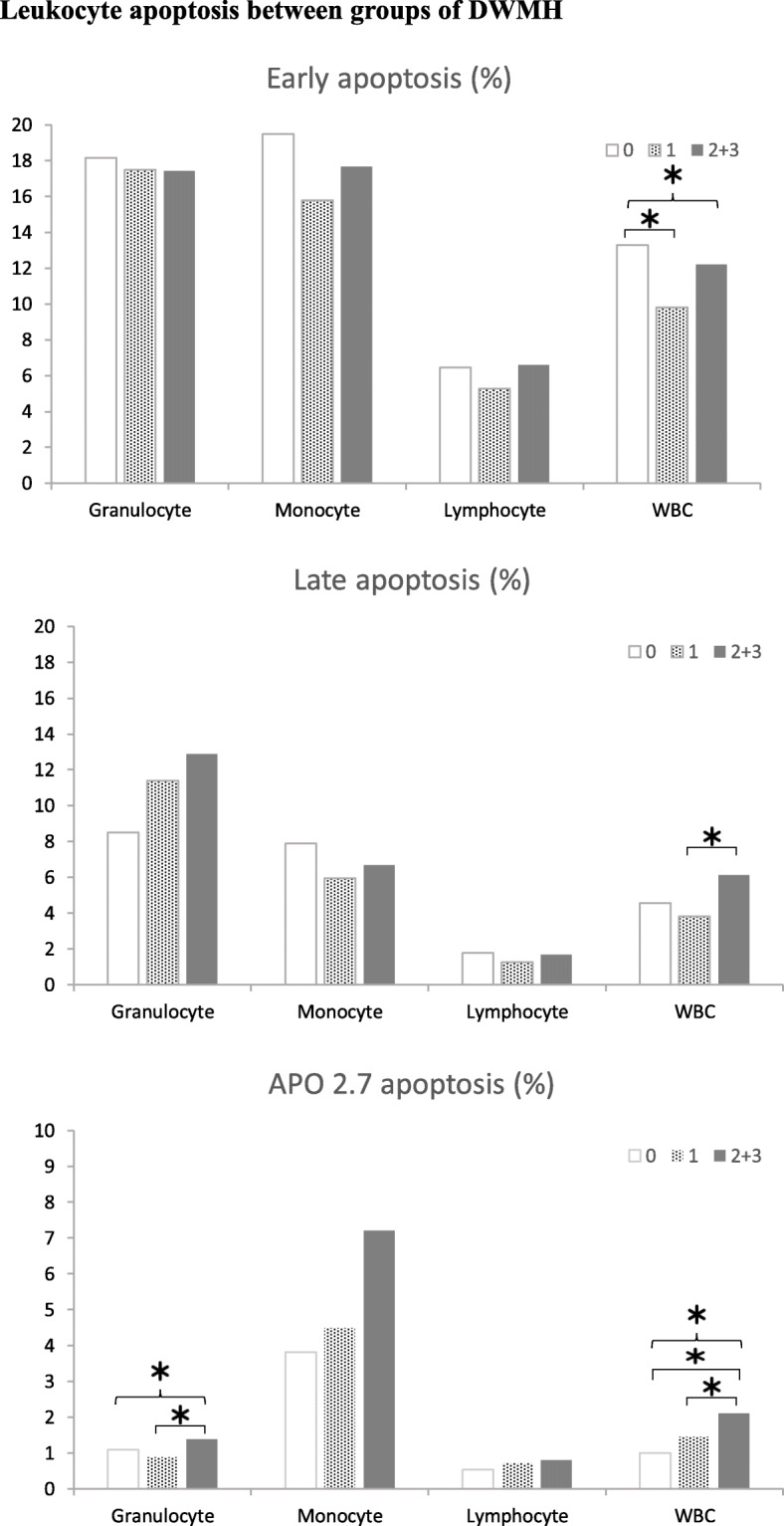
Leukocyte apoptosis between groups of DWMH. The percentages of (**a**) early apoptosis, (**b**) late apoptosis, and (**c**) APO 2.7 apoptosis in patients with normal, mild, and moderate to severe deep white matter (DWM) lesions. Apoptotic cells were defined as APO 2.7-positive. Leucocyte subtypes were identified according to their CD45 expression intensity. The presence of late apoptotic cells meant that the cell membrane integrity was disrupted. The presence of early apoptotic cells indicated that the apoptotic change was early and still reversible. Early apoptosis of total leucocytes was lower in patients with advanced DWM lesions. However, the late apoptosis of total leucocytes and APO 2.7 apoptosis of granulocytes and total leucocytes were significantly higher in patients with advanced DWM lesions compared with normal lesions. Data are presented as mean ± standard deviation. * *P* < 0.05 in group comparisons. DMWH, deep white matter hyperintensities

**Fig. 2:**
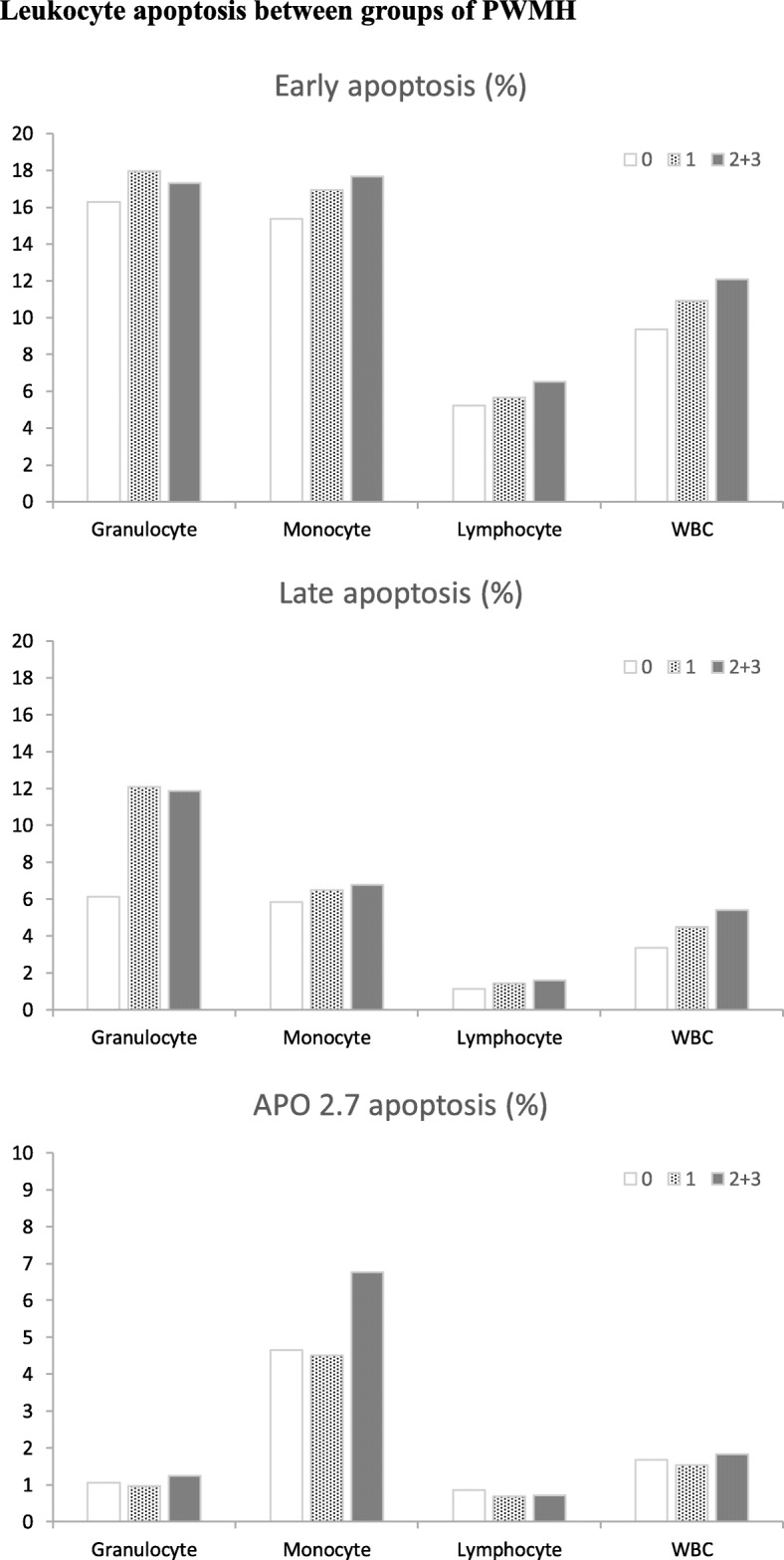
Leukocyte apoptosis between groups of PWMH. The percentages of (**a**) early apoptosis, (**b**) late apoptosis, and (**c**) APO 2.7 apoptosis in patients with normal, mild, and moderate to severe periventricular white matter (PWM) lesions. There was no significant difference in group comparisons of normal and different gradings of PWM lesions. PMWH, periventricular white matter hyperintensities

As shown in Fig. 1, there were significant differences in the percentages of total leucocyte early apoptosis, granulocyte APO 2.7, and total leucocyte APO 2.7 in PD patients with different DWMH gradings (*p*=0.018, 0.033, 0.049). A significant difference was revealed in the percentage of total leucocyte early apoptosis between patients with normal and mild DWMHs (*p*=0.015). There was a significant difference in the percentage of total leucocyte late apoptosis between patients with mild and moderate to severe DWMH damages (*p*=0.022). Significant differences were seen in the percentages of granulocyte and total leucocyte APO 2.7 between patients with mild and moderate to severe DWMH damage (*p*=0.004, 0.016). However, no significant difference was found in all detected oxidative stress biomarkers among PD patients with different PWMH gradings (Fig. 2).

## Discussion

We investigated the oxidative stress biomarkers and cognitive functions in patients with PD with PWMH or DWMH lesions and identified the changes in oxidative stress biomarkers and cognitive deficits between PD patients with different PWMH or DWMH gradings. According to the results, the percentages of granulocyte and total leucocyte APO 2.7 were significantly higher in patients with PD with more severe DWMH. In addition, the percentages of early apoptosis of total leucocytes were also significantly lower in patients with DWMH lesions than in those without DWMH lesions. Regarding cognitive performance, there were significant deficits in domains of cognitive function in patients with PD having more severe PWMH lesions. To the best of our knowledge, this is the first study to report the changes in the circulating oxidative biomarkers in PD patients with different PWM or DWM alternations.

Small vessel disease is common, causing substantial cognitive and physical disabilities in older peoples [41, 42]. One of its main imaging features is white matter hyperintensities [43]. The possible associated vascular risk factors include hypertension, diabetes mellitus, hypercholesterolemia and smoking [44–46], though the clear mechanisms are largely unknown. Furthermore, periventricular and deep white matter injuries were reported to be associated with different cardiovascular factors and may have different aetiologies in the elderly. Studies have proposed that DWMH is associated with higher body mass index but not arterial pressure, while PWMH may be linked to higher arterial pressure but not with BMI [26, 29]. For the pathogenesis of white matter changes with ageing, some studies hypothesised more hypoxic/ischaemic damage in DWMHs, but greater inflammatory/metabolic components in PWMH [39, 47]. For PD with white matter alternations, we investigated the changes in oxidative stress biomarkers and found higher percentages of granulocyte APO 2.7 and total leucocyte APO 2.7 in patients with moderate to severe DWMH damage. However, higher percentages of early apoptosis of total leucocytes was found in patients without DWMH damage in this study, and we considered it might be those PD patients without DWMH already experienced the early apoptosis before brain deep white matter structural changes detected by brain imaging. The real pathophysiology was still unknown, and it requires further study to explore. There were no significantly higher oxidative stress biomarkers in patients with PD having more severe PWM lesions. The findings for oxidative biomarkers in PD with different white matter injuries were not similar to those in previous reports on elderly individuals with DWMH or PWMH. The reason could be related to the presence of hypoxia in PD, which related to respiratory dysfunction, including restrictive changes, upper airway obstruction, abnormal ventilatory drive, and response to medications [48], contributed by deep white matter damage and associated with increasing circulating inflammatory markers in PD. This finding could be similar to that of hypoxia in patients with severe obstructive sleep apnoea who had impaired white matter integrity due to increased systemic inflammation [22].

In the elderly, worse cognition domains, especially in memory and executive function/processing speed, were associated with PWMH rather than DWMH lesions [30]. PWMH lesions interfere with long connections, leading to negative impacts on mostly cognitive domains. Deep white matter damage is responsible for short connections that are less associated with cognitive performance but may play an important role in motor dysfunction [26]. For patients with PD, we also found that PD with more PWMH lesions had significant declines in more cognitive domains. Furthermore, we found that PD patients with more severe PWM damage had a tendency for deficits in executive function and a significant decrease in visual-spatial function compared with PD patients without PWMH lesions. The explanation for this finding is that executive and visual-spatial functions are a more complex process involving more brain areas [49], and therefore, severe PWM injuries may disrupt more long connections, leading to an obvious deficit. Additionally, PD patients with more severe deep white matter injuries also had reduced attention and memory functions in our study. Further studies are needed to identify supporting brain regions of white matter associated with cognitive functions in patients with PD.

The limitations of this study are as follows. First, all PD patients with white matter lesions were recruited from one medical centre. Second, no normal individuals were included in the control group for excluding white matter alternations due to ageing process. Third, most PD patients with less disease severity were enrolled in this study, and therefore could not represent the general population of PD. Fourth, there is a borderline significant difference in hypertension between mild and moderate to severe gradings of DWMH lesions. Further correlation between hypertension, oxidative stress and cognitive performance were not conducted in our study. In the future, we hope to conduct more studies to enrol early PD patients without white matter injury and to conduct a longitudinal study to observe their neuro-structural and cognitive changes and to determine the role of neuroinflammation in PD with white matter injuries.

## Conclusion

In summary, we found significant alternations in some oxidative stress biomarkers only in PD patients with DWMH, but not PWMH. Systemic inflammatory responses may contribute to deep white matter damage in PD. In patients with PD, more severe periventricular white matter injury was associated with more cognitive deficits, some of which were also found in patients with worse deep white matter lesions. Clinically, this study suggests practical interventions in PD to inhibit the inflammatory response and prevent or impede the progression of white matter injury related to cognitive impairments.

## Data Availability

The datasets generated and analysed during the current study are not publicly available due to the ethical reasons but are available from the corresponding author on reasonable request.

## Abbreviations

CASI: Cognitive Ability Screening Instrument
DWMH: Deep white matter hyperintensities
HYSS: Hoehn and Yahr Staging Scale
MMSE: Mini-Mental State Examination
MRI: Magnetic resonance imaging
PD: Parkinson’s disease
PWMH: Periventricular white matter hyperintensities
